# Changes in fatigue symptoms following an exercise-based rehabilitation programme for patients with long COVID

**DOI:** 10.1183/23120541.00089-2024

**Published:** 2024-07-22

**Authors:** Enya Daynes, Molly M. Baldwin, Matthew Annals, Nikki Gardiner, Emma Chaplin, Sarah Ward, Neil J. Greening, Rachael A. Evans, Sally J. Singh

**Affiliations:** 1 Centre for Exercise and Rehabilitation Sciences, NIHR Leicester Biomedical Research Centre Respiratory, Leicester, UK; 2 Department of Respiratory Sciences, University of Leicester, Leicester, UK

## Abstract

**Background:**

There is evidence to support COVID-19 rehabilitation programmes improving persistent COVID-19 symptoms; however, there is concern that therapies that include an exercise component may increase fatigue and post-exertional symptom exacerbation (PESE). The objectives of the present study were to determine the effect of a 6-week COVID-19 rehabilitation programme on fatigue and PESE in individuals with ongoing COVID-19 symptoms.

**Methods:**

After a routine medical assessment, individuals with persistent COVID-19 symptoms were enrolled on a 6-week COVID-19 specific rehabilitation programme. The programme included symptom-titrated exercise, education and self-management advice. Fatigue was assessed pre- and post-programme using the Functional Assessment Chronic Illness Therapy Fatigue questionnaire (FACIT). Exercise capacity (Incremental and Endurance Shuttle Walking Test (ISWT and ESWT)) and PESE (DePaul Symptom Questionnaire (DSQ)) were also assessed pre- and post-programme. Composite scores were calculated for the frequency and severity domains of the DSQ.

**Results:**

148 patients (median (IQR) age 59 (49–72) years, 82 (55%) female, 81 (54%) hospitalised) completed the COVID-19 rehabilitation programme. FACIT score was reduced pre- to post-programme by a mean (CI) change of −5 (−7– −4); p<0.01. Exercise capacity increased by 82 (65–99) m for the ISWT and 398 (333–462) s for the ESWT (n=148). PESE was assessed in 44 patients. The DSQ frequency and severity composite score improved by 20 (13–28) and 19 (13–26) points, respectively (p<0.01, n=44).

**Conclusion:**

These data demonstrate the potential benefits of a COVID-19 rehabilitation programme in improving fatigue, exercise capacity and symptom exacerbation in those with persistent COVID-19 symptoms.

## Shareable abstract

**Rehabilitation can improve fatigue and post-exertional symptom exacerbation for those with long COVID** https://bit.ly/4aHQVnD

## Introduction

SARS-CoV-2 is a virus with multisystem effects that predominantly affects the respiratory system and, prior to vaccination or exposure, commonly results in pneumonitis, potentially resulting in hospitalisation and ventilatory support. Recovery from COVID-19 can be prolonged and incomplete, often taking up to 12 weeks [1]. However, ongoing symptoms are common, with up to 70% of patients hospitalised due to COVID-19 experiencing ongoing symptoms for at least 12 months post-hospital discharge [1, 2]. Individuals who managed their acute infection in the community often develop similar long-term symptoms, with 1.8 million people in the UK experiencing long COVID symptoms (2% of the total population) [3, 4]. Symptoms lasting >12 weeks after acute COVID-19 are often termed “post-COVID syndrome” or “long COVID” [3]. The most common persistent symptoms include fatigue, breathlessness, pain, cognitive impairment and reduced functional capacity [1, 3]. Owing to the volume of individuals affected and magnitude of symptomatic burden experienced, effective rehabilitation strategies are needed that attenuate persistent COVID-19 symptoms.

Fatigue is a complex, multidimensional symptom commonly present in long-term conditions, such as chronic respiratory diseases, as well as following acute respiratory conditions such as pneumonia [5, 6]. Similarly, post-viral fatigue is a phenomenon that persists after viral infection and has been demonstrated in >15% of people following Middle East Respiratory Syndrome (MERS) and Severe Acute Respiratory Syndrome (SARS) infections and lasts between 6 weeks and 39 months [7]. The exact prevalence of fatigue following COVID-19 is difficult to determine; however, generally it is the most common reported symptom [2]. The factors contributing to fatigue are complex and cover central, peripheral and psychological factors, which can make the diagnosis and management of fatigue following COVID-19 difficult [8]. Owing to the relative contribution of the influencing factors and causes, it is likely that individuals will require personalised clinical management strategies.

Tailored rehabilitation programmes, containing symptom-titrated exercise and education/advice on symptom management strategies, have demonstrated improvements in fatigue in those with chronic respiratory conditions and have shown promise following COVID-19 [9–11]. However, there is reported concern around the potential contribution of exercise to worsening symptoms for those with long COVID [12]. Post-exertional symptom exacerbation (PESE) refers to a worsening of symptoms following activity or exercise that exceeds the expected response to the activity [12]. These symptoms can include, but are not limited to, fatigue, pain and cognitive impairment and often occur between 24 and 72 hours after the activity. PESE and/or post-exertional malaise (PEM) can be monitored using the DePaul Symptom Questionnaire (DSQ). Although it does not offer a diagnosis as a standalone tool, the DSQ may be useful in assessing worsening of symptoms following rehabilitation programmes [13]. This questionnaire has been used in patients with chronic fatigue syndrome/myalgia encephalomyelitis [14], but there are limited data in people with long COVID, particularly over multiple time points or in response to an intervention.

The aims of this study were to explore the presence and severity of fatigue and fatigue-related symptoms associated with PESE in individuals with long COVID, and determine the effect of a COVID-19 rehabilitation programme on such symptoms.

## Methods

### Study design

This was a prospective observational cohort study conducted in the clinical COVID rehabilitation service at University Hospitals of Leicester NHS Trust. The cohort included individuals who were hospitalised during their index COVID-19 infection and those who managed this infection in the community. Individuals were eligible if they self-reported ongoing symptoms beyond 12 weeks of their acute COVID-19 infection that affected their daily activities (*e.g.* breathlessness, fatigue, weakness, functional limitation, *etc.*). Individuals were excluded if they demonstrated acute symptoms, were not medically stable or had symptoms that were deemed not modifiable by a rehabilitation programme (*e.g.* loss of taste only). Individuals hospitalised from COVID-19 were referred through a discharge follow-up pathway that included specialist review at a COVID-19 clinic. Individuals managed in the community were referred by their general practitioner to the specialist consultant-led COVID-19 clinic prior to enrolment in rehabilitation. All individuals were screened for unexplained symptoms that required further investigation by their clinician. All patients had a comprehensive assessment, which included exploring the individual's needs and goals, with a healthcare professional in the rehabilitation team prior to starting the rehabilitation programme. The study was approved as a substudy amendment by the National Health Service Research Ethics Committee (reference 17/EM/0156) and registered through the ISRCTN (ISRCTN45695543).

### Intervention

The COVID-19 rehabilitation programme was 6 weeks in duration and consisted of two supervised sessions per week of symptom-titrated exercise followed by an education component. The sessions lasted ∼90 min (60 min of exercise, with suitable rest periods, and 30 min of education) and participants were encouraged to work at their own pace. Exercise was modified in line with patient symptoms. The symptom-titrated exercise comprised of aerobic exercise (walking/treadmill based) and resistance exercise training of the upper and lower limbs. The intensity of the aerobic exercise was determined through the Incremental Shuttle Walking Test (ISWT), with training commenced at 80% of maximum speed when feasible. Resistance training was prescribed at a relative intensity, ensuring participants could complete approximately three sets of 10 repetitions. This was progressed by increasing weights once participants reported a reduction in the rate of perceived exertion. At each session the Borg breathlessness scale and rate of perceived exertion were used alongside self-reported symptoms from the previous days (including fatigue) to explore response to exercise and determine progression in line with patient symptoms.

Educational sessions were guided by participants, tailored to their individual needs, and supported by handouts from the Your COVID Recovery website (www.yourcovidrecovery.nhs.uk available until 30 April 2024). Self-management strategies were discussed within the group and reinforced on a one-to-one basis throughout the programme. Topics covered included self-management strategies around activities of daily living, pacing, prioritising, managing cognitive impairment, breathlessness, cough, fatigue, fear and anxiety, taste and smell, eating well, getting moving again, sleeping well, and returning to work. These strategies were discussed within the group and reinforced on a one-to-one basis throughout the programme.

Before and after intervention, participants completed measures of exercise capacity using the ISWT and Endurance Shuttle Walking Test (ESWT) [15, 16]. Fatigue was assessed using Functional Assessment of Chronic Illness Therapy Fatigue scale (FACIT) [17]. Mood disturbance was measured using Hospital Anxiety and Depression Scale (HADS) [18], and general health-related quality of life was assessed using the EuroQol 5 domains (EQ5D) thermometer score, exploring health today on a 0–100 scale [19]. The COPD Assessment Test (CAT) was used to assess common symptoms and has shown validity in patients with COVID-19 [20]. The DSQ [13] was also completed by a subgroup of patients. The DSQ was added in response to advice from patient representatives and was therefore performed on consecutive patients that attended an assessment between May and December 2021. This questionnaire explores symptom exacerbation in relation to severity and frequency of physical and cognitive fatigue; however it is not used as a diagnostic tool. In line with gold standards, the ISWT and ESWT were completed on a 10-m course, and a familiarisation test was performed at baseline for the ISWT [21].

### Statistical analysis

Data were analysed using SPSS v25. The sample included all those attending rehabilitation that completed the measures of interest (FACIT and DSQ). Individuals were considered to have completed the rehabilitation programme if they attended at least eight out of 12 scheduled sessions. Individuals were grouped as having “severe fatigue” or “not severe fatigue” based on the FACIT cut-off <30 points or ≥30 points, respectively [17]. The DSQ was analysed as recommended using a composite score for questions 1–5 on severity and frequency of symptoms, calculated using the following equation: (mean of questions 1–5) × 25 [14]. Changes pre- and post-rehabilitation were compared using paired t-test for parametric continuous data, signed-rank for non-parametric continuous data and chi-squared test for categorical variables. The number of participants meeting the minimal important difference (MID) for all outcomes was explored using known MID in chronic respiratory disease, or where available in COVID-19; this included the CAT (2 points), HADS (1.5 points), FACIT (3 points), ISWT (35 m) and ESWT (174 s) [22–28].

## Results

### Baseline characteristics

The COVID-19 rehabilitation programme was completed by 170 individuals between September 2020 and December 2021. Patient characteristics are presented in table 1. Discharge assessment data were available for 148 patients; 22 patients did not complete their discharge assessment due to the following reasons: lost to follow-up (n=6), other coexisting health condition (n=4), work/social commitments (n=4), shielding due to rising number of COVID-19 cases (n=1), completed the programme but did not complete the questionnaires (n=6) and unwell at the time of discharge assessment (n=1). At the time of assessment, no patients had a known clinical diagnosis of PESE or PEM.

**TABLE 1:** Characteristics of patients (n=148)

**Age years**	59 (49–72)
**Sex, female**	82 (60)
**Ethnicity, white British**	104 (64)
**Number of patients hospitalised due to COVID-19**	81 (54)
Length of hospital admission, days	7 (2–17)
Time since hospitalisation, days	154 (84–233)
**No. of comorbidities (n=139)**	1 (0–2)
0	48 (35)
1	39 (28)
≥2	52 (42)

Data are presented as median (IQR) or n (%).

### Changes following a rehabilitation programme

**FIGURE 1:**
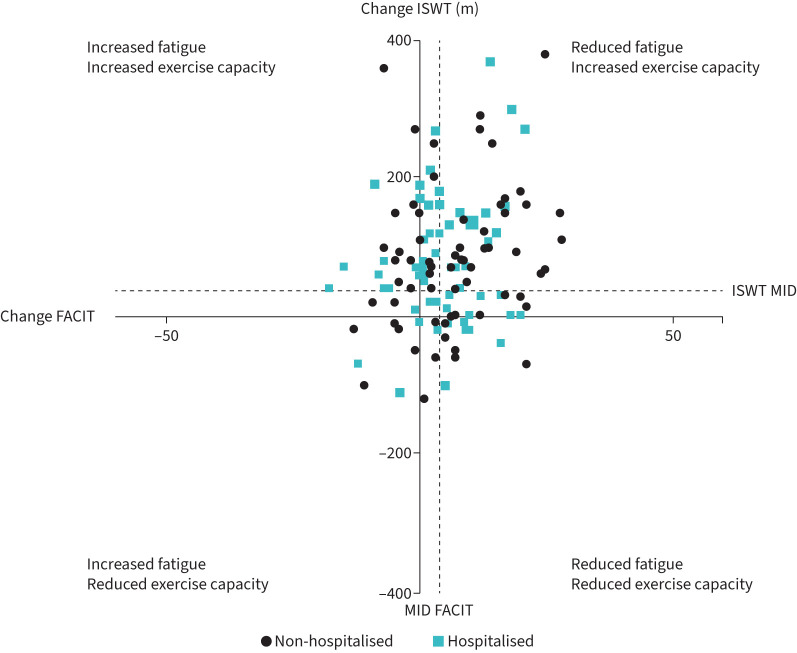
Changes in ISWT and FACIT following rehabilitation. ISWT: Incremental Shuttle Walking Test; FACIT: Functional Assessment of Chronic Illness Therapy Fatigue scale.

The mean±sd FACIT score at baseline was 25±12 (table 2). Pre- to post-programme, there were improvements in the CAT (mean±sd 3±6), HADS Anxiety (HADS-A) (0.86±3.44), HADS Depression (HADS-D) (0.98±3.74) and EQ5D thermometer (6±19) (p<0.01; figure 1, table 2). Exercise capacity also improved pre- to post-programme, with the ISWT and ESWT increasing by a mean of 85±98 m and 400±385 s, respectively (table 2).

**TABLE 2:** Measures of fatigue, exercise performance, health-related quality of life, anxiety and depression pre- and post-rehabilitation

	Pre-rehabilitation, mean±sd	Post-rehabilitation, mean±sd	Change, mean (CI)	Achieving MID, n (%)
**FACIT (n=148)**	25±12	30±12	5 (4–7)**	89 (60) [22]
**Incremental Shuttle Walking Test m (n=148)**	332±183	414±189	82 (65–99)**	114 (77) [24]
**Endurance Shuttle Walking Test s (n=140)**	256±207	654±431	398 (333–462)**	95 (68) [25]
**COPD Assessment Test (n=147)**	18±7	15±7	3 (2–4)**	90 (61) [26]
**HADS-A (n=145)**	8.42±4.84	7.56±4.63	0.86 (0.29–1.43)**	20 (14) [27]
**HADS-D (n=145)**	7.60±4.47	6.62±4.71	0.98 (0.97–1.59)**	25 (17) [27]
**EQ5D thermometer VAS 0–100 (n=92)**	59±19	66±20	7 (3–9)**	39 (42) [28]

MID: minimal important difference; FACIT: Functional Assessment of Chronic Illness Therapy; HADS: Hospital Anxiety and Depression Scale; A: Anxiety; D: Depression; EQ5D: Euroqol 5 domain; VAS: visual analogue scale. **p<0.01.

### Fatigue

**FIGURE 2:**
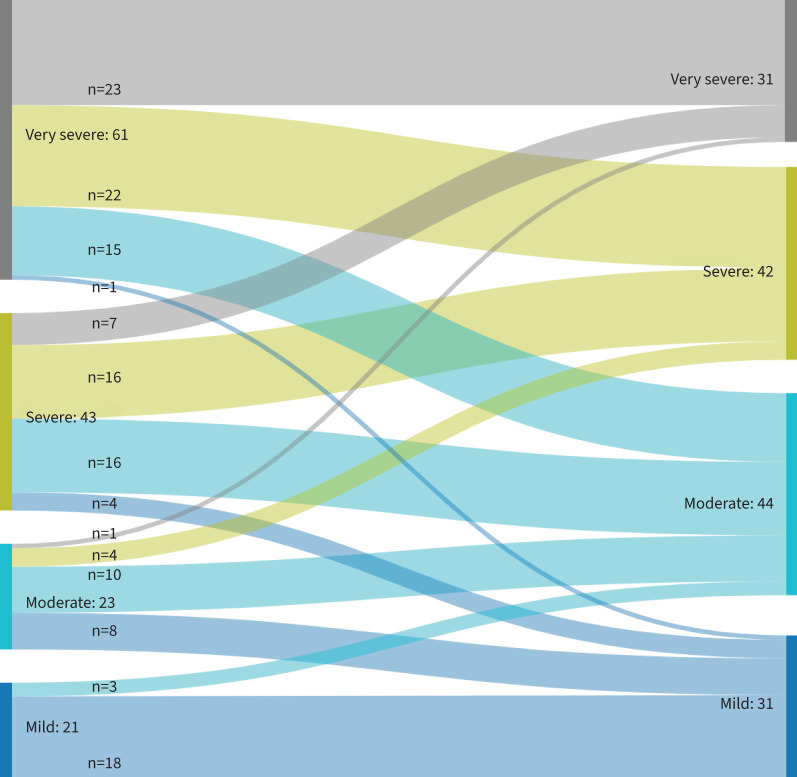
Sankey diagram of changes in FACIT following rehabilitation. Very severe 0–20, severe 21–30, moderate 31–40, mild 41–52. FACIT: Functional Assessment of Chronic Illness Therapy Fatigue scale.

FACIT score increased pre- to post-programme by a mean of 5±9 points (table 2). 89 (60%) patients improved their score pre- to post-programme by the MID (3 points; figure 2) [23]. There were no differences in ISWT, ESWT or CAT in those who improved their FACIT score by the known MID compared to those who did not improve (p>0.05).

When individuals were categorised as having either severe or not severe fatigue based on a FACIT score of <30 or ≥30 points, 104 (70%) individuals had severe fatigue pre-programme. FACIT improved by 7±9 and 2±7 points for severe fatigue and not severe fatigue, respectively (p=0.03). There were no differences between those with severe fatigue and those without severe fatigue in relation to the change in ISWT (mean±sd difference 23±18 m, p=0.13), ESWT (82±88 s, p=0.35), CAT (0.42±1.07, p=0.70), HADS-A (0.57±0.62, p=0.36), HADS-D (0.33±0.65, p=0.36) or EQ5D thermometer (3.42±3.40, p=0.32).

FACIT score did not improve after the rehabilitation programme by a magnitude of ≥1 point in 30 (20%) individuals; however, ISWT distance and ESWT duration increased in these individuals by 82±109 m and 356±473 s, respectively (p<0.01). Of those that had a worse FACIT score, only one participant did not see an improvement in ISWT distance or ESWT time. This was explored and may be due to a musculoskeletal injury sustained prior to the participant's discharge appointment impairing performance.

The number of individuals with very severe (score 0–20), severe (21–30), moderate (31–40) and mild fatigue (41–52) pre- and post-programme is displayed in figure 2.

### Post-exertional symptom exacerbation

A subset of consecutive individuals attending the service between May and December 2021 (n=44) completed the DSQ pre- and post-rehabilitation. There is a statistically significant, moderate correlation between the FACIT and DSQ for frequency (−0.56, p<0.01) and severity (−0.58, p<0.01), respectively. DSQ frequency and severity composite scores improved pre- to post-programme by 20±24 and 19±21, respectively (p<0.01; table 3). There was also an improvement in questions 6, 8 and 10, which relate to recovery after activity, fatigue after mental effort/concentration and avoiding exercise due to symptom exacerbation, respectively (supplementary tables S1 and S2).

**TABLE 3:** DePaul Symptom Questionnaire composite score for frequency and severity pre- and post-rehabilitation (n=44)

	Pre-rehabilitation, mean±sd	Post-rehabilitation, mean±sd	Change, mean (CI)	p-value
**Composite score frequency**	86±23	65±24	20 (13–28)	p<0.01
**Composite score severity**	84±22	65±25	19 (13–26)	p<0.01

## Discussion

This is the first study to investigate the effect of a 6-week symptom-titrated exercise-based rehabilitation programme on fatigue and PESE in individuals with long COVID. Our data demonstrate that the rehabilitation programme was effective at decreasing anxiety and depression, and increasing exercise capacity and health-related quality of life. However, the primary novel findings were that the programme simultaneously reduced fatigue, and the frequency and severity of PESE symptoms in this clinical cohort.

Fatigue has been consistently reported as one of the most common and troublesome symptoms following COVID-19. Rehabilitation strategies have demonstrated improvements in fatigue in other conditions such as COPD; however there is little literature exploring the impact of these strategies on fatigue in those with long COVID. The present study suggests that symptom-titrated exercise delivered with symptom management strategies can be used to ameliorate fatigue in long COVID, though this does not account for natural recovery. Whilst we wait for a randomised control trial to assess the effectiveness of rehabilitation on fatigue in this cohort [29], the present data are encouraging, and highlight the potential of this intervention to reduce symptomatic burden.

This programme is based on a pulmonary rehabilitation programme for chronic respiratory diseases where there is a wealth of positive literature. Both the pulmonary and COVID rehabilitation programmes provide a tailored and individually prescribed exercise component and, therefore, the modifications largely related to the educational component, with emphasis on managing and monitoring fatigue. A primary objective of clinical rehabilitation programmes is to increase exercise capacity as it is a strong predictor of morbidity and mortality, and correlates with health-related quality of life in individuals with respiratory conditions. The rehabilitation strategy employed in this study effectively increased exercise capacity (ISWT distance), with a similar increase seen in those with and without severe fatigue. The results of this study are comparable to the literature where rehabilitation/exercise-based interventions have shown improvements in exercise capacity and fatigue within long COVID [30–32]. This suggests that the presence of severe fatigue does not impair the adaptive response to exercise training. It also shows that individuals post-programme were able to do more before they induced fatigue limitations, which was notably less severe. Treadmill speed was initially set at 80% of maximum (where it was feasible to do so); however, the ISWT provides a predicted peak oxygen uptake (*V*′O_2_), and therefore it is possible that these individuals are performing at a lower intensity, though this did augment benefits in exercise capacity and symptoms. Thus, it is an appropriate method for prescribing intensity, in the absence of a cardiopulmonary exercise test.

Treating fatigue is complex as there are potentially multiple mechanisms interacting, and determining which mechanism is at play for each individual is challenging. Exercise and physical activity have demonstrated improvements in immune cell function and pro-inflammatory cytokine release, even with low levels of activity, whilst also reversing the effects of deconditioning [33–39]. It is important that these modalities are individually tailored in order to prevent a worsening of symptoms, and combined with strategies such as pacing and prioritising may provide additional benefit in alleviating fatigue.

The DSQ indicates whether PESE or PEM is present; it is not a diagnostic tool without a clinician assessment. Prior to enrolment on the programme, all participants were seen by a medical professional and referred for COVID-19 rehabilitation. No individuals in this cohort had a known clinical diagnosis of PEM; however, many experienced PESE as defined by the DSQ, though they did not demonstrate severe symptom exacerbation during the programme. It is likely that individuals with severe PEM resulting in disability (*i.e.* bedridden) were referred to other services instead of COVID-19 rehabilitation. The rehabilitation programme employed in this study improved both the frequency and severity of PESE symptoms in individuals with persistent COVID-19 symptoms. In line with this result, a 6-week World Health Organization Borg CR-10 pacing protocol, including walking-based exercise, reduced the number of PESE episodes in individuals with long COVID from 3.4 to 1.1 episodes per week [40]. The clinical context of the changes in PESE symptoms seen in this study is difficult to determine, particularly in the absence of a known MID for the DSQ in long COVID. However, irrespective of whether the observed reduction in PESE symptoms translates to a clinical benefit, these data suggest that individuals with PESE symptoms may participate in symptom-titrated exercise-based rehabilitation, and induce improvements in fatigue and exercise capacity, without augmenting PESE symptoms, thus indicating the potential of exercise-based rehabilitation programmes in the management of long COVID in those with PESE symptoms. However, further studies are needed to confirm this.

### Clinical implications

Concerns have been raised that exercise-based rehabilitation programmes may augment symptoms of fatigue and PESE in individuals with post-COVID-19 conditions. We observed a reduction in fatigue and PESE pre- to post-programme in conjunction with an increase in exercise capacity, thus highlighting the potential benefit of managing symptoms in individuals with long COVID with exercise-based rehabilitation strategies; however, these findings need to be confirmed by a randomised controlled trial. Long COVID rehabilitation programmes may require some modifications or adaptations in order to ensure they are appropriate for this population. Notably this includes a thorough medical assessment to exclude any risk factors, adjustments to educational component and close monitoring of fatigue symptoms [41].

### Strengths and limitations

There are several strengths and limitations to this study. This study is representative of the long COVID population requiring specialist care and provides an exploration of the effect of a rehabilitation programme on fatigue and PESE. This is a large cohort study exploring fatigue and PESE; however, there is an absence of a control group, and therefore it does not account for natural recovery. However, the literature has demonstrated minimal improvements in exercise capacity and health status between 5 and 12 months post-acute infection in an observational cohort study [2]. The FACIT questionnaire was used to assess fatigue, and therefore these results are not generalisable to other forms of fatigue (such as cognitive fatigue). The DSQ was completed by a small subset of consecutive individuals between May and December 2021 following advice from expert clinicians and patient advisory groups to include this questionnaire. This study does not aim to offer a formal diagnosis of PEM or PESE but to report symptoms in response to exercise before and after a rehabilitation programme. This may support identification of patients not responding to the intervention and who require referral to other services, though this was not demonstrated in this cohort. The DSQ can offer insight into these symptoms and can be used in other programmes or other services to explore the potential prevalence and impact of PESE or PEM. The time since COVID infection in non-hospitalised patients and level of respiratory support during hospitalisation was not accurately reported, and therefore it is difficult to establish the length of time people have been living with long COVID; however, cohort studies have demonstrated little improvement in symptoms over 12 months [2].

### Conclusion

This study demonstrates the potential benefits of a COVID rehabilitation programme in improving fatigue, quality of life and exercise capacity in a large cohort of patients displaying severe fatigue. These data support the offer of symptom-titrated exercise training following COVID-19 with supported monitoring of symptom exacerbation.

## Supplementary material

10.1183/23120541.00089-2024.Supp1 **Please note:** supplementary material is not edited by the Editorial Office, and is uploaded as it has been supplied by the author.
Supplementary material 00089-2024.SUPPLEMENT

## Data Availability

Group data are available upon reasonable request from the corresponding author.
