# Supplementary material for: Changes in fatigue symptoms following an exercise-based rehabilitation programme for patients with long COVID
Source: ERJ Open Res. 2024 Jul 22;10(4):00089-2024. doi: 10.1183/23120541.00089-2024 (PMC11261384; doi:10.1183/23120541.00089-2024)
Supplement: Supplementary file 1 [file 00089-2024.SUPPLEMENT.pdf]

Supplementary material

S1 Changes in outcomes for the subgroup that completed the DSQ questionnaire.

	Pre	Post	Change
FACIT n=35	19.53[8.40]	27.74[11.07]	8.21[10.51]p<0.01
ISWT n=28	317.14[167.09]	407.5[172.04]	90.36[100.94] p<0.01
ESWT n=37	238.30[142.65]	536.47[369.42]	298.17[321.50] p<0.01
CAT n=41	19.10[7.95]	16.02[9.68]	3.07[8.09] p=0.02
HADS-A n=43	9.42[5.04]	7.95[3.91]	1.47[4.11] p=0.02
HADS-D n=43	8.14[4.32]	7.28[5.46]	0.86[4.14] p=0.18
EQ5D thermometer n=36	56.81[15.98]	63.17[20.20]	6.36[19.78] p=0.06

S2 Analysis of question six to ten of the DePauls Symptom Questionnaire.

Would you recover within one hour of the activity ending?	23(52%) no	22(50%) no	p<0.01
Do you experience worsening of your fatigue after engaging in minimal effort?	8(19%) no	21(48%)no	p=0.08
Do you experience worsening of fatigue after engaging in mental effort?	6(14%) no	14(32%) no	p=0.01
If you feel worse after activities, how long does this last?	1= 7 (17%), 2= 16 (38%), 3=11(26%), 4=0, 5=2(5%), 6=6(14%)	1=12(28%), 2=14(33%), 3=9(21%), 4=0, 5=1(3%), 6=7(16%)	p=0.11
If you do not exercise, is it because it makes your symptoms worse?	11(26%) no	25(60%) no	p=0.01
